# Functional roles of the [2Fe-2S] clusters in *Synechocystis* PCC 6803 Hox [NiFe]-hydrogenase reactivity with ferredoxins

**DOI:** 10.1016/j.jbc.2024.107936

**Published:** 2024-10-28

**Authors:** Matthew R. Blahut, Michael E. Dawson, Effie C. Kisgeropoulos, Anastasia E. Ledinina, David W. Mulder, Paul W. King

**Affiliations:** Biosciences Center, National Renewable Energy Lab, Golden, Colorado, USA

**Keywords:** *Synechocystis*, iron-sulfur cluster, diaphorase, ferredoxin, [NiFe]-hydrogenase, EPR, square wave voltammetry, redox potential, electron transfer

## Abstract

The HoxEFUYH complex of *Synechocystis* PCC 6803 (*S*. 6803) consists of a HoxEFU ferredoxin:NAD(P)H oxidoreductase subcomplex and a HoxYH [NiFe]-hydrogenase subcomplex that catalyzes reversible H_2_ oxidation. Prior studies have suggested that the presence of HoxE is required for reactivity with ferredoxin; however, it is unknown how HoxE is functionally integrated into the electron transfer network of the HoxEFU:ferredoxin complex. Deciphering electron transfer pathways is challenged by the rich iron-sulfur cluster content of HoxEFU, which includes a [2Fe-2S] cluster in each subunit, along with multiple [4Fe-4S] clusters and a flavin cofactor. To resolve the role of HoxE, we determined the biophysical and thermodynamic properties of each [2Fe-2S] cluster in HoxEFU using steady-state and potentiometric EPR analysis in combination with square wave voltammetry (SWV). The temperature-dependence of the EPR signal for HoxE confirmed the coordination of a single [2Fe-2S] cluster that was shown by SWV to have an *E*_m_ = −424 mV (*versus* SHE). Strikingly, when the *E*_m_ of the HoxE [2Fe-2S] cluster was analyzed in HoxEFU titrations, it was shifted by >100 mV to an *E*_m_ < −525 mV (*versus* SHE). EPR titrations of HoxEFU gave an *E*_m_ value for the [2Fe-2S] cluster of HoxF, *E*_m_ = −419 mV and HoxU, *E*_m_ = −349 mV. These values were used to re-analyze the diaphorase kinetics in reactions performed with ferredoxins with varying *E*_m_’s. The results are formulated into a model of HoxEFU:ferredoxin reactivity and the role of HoxE in mediating electron transfer within the HoxEFU:ferredoxin complex.

Understanding the function of proteins that mediate electron transfer and reduction–oxidation reactions is crucial to the development of renewable energy and biofuel technologies based on utilization of photosynthetic organisms. One group of enzymes that have an instrumental role in these reactions is hydrogenases, which catalyze the reversible oxidation of molecular hydrogen (1, 2). A major class of hydrogenases found in cyanobacteria are the multi-subunit, bidirectional Hox [NiFe]-hydrogenases (Group 3d) (3, 4). Hox [NiFe]-hydrogenase from cyanobacteria consists of five subunits, HoxE, HoxF, HoxU, HoxY and HoxH and performs key functions in mediating redox biochemistry during photosynthetic growth (5, 6, 7). For example, the Hox [NiFe]-hydrogenase of *Synechocystis* sp. PCC 6803 (*S*. 6803), a model photosynthetic organism, catalyzes H_2_ activation coupled to the NAD(P) (H) and ferredoxin electron carrier pools (6, 8). Thus, understanding the properties and function of HoxEFUYH is important to enabling the bioengineering of *S*. 6803 and related cyanobacteria as model systems for photobiological hydrogen-production (9).

The HoxEFUYH complex is composed of two functionally independent sub-complexes, HoxYH and HoxEFU (10, 11, 12, 13). HoxYH, which functions in catalytic H_2_ activation, consists of a small subunit, HoxY, and a large, [NiFe] active-site containing subunit, HoxH (14). The HoxEFU sub-complex, which catalyzes diaphorase reactivity (6, 7, 15, 16), binds one flavin mononucleotide on HoxF and 3x[2Fe-2S] and 4x[4Fe-4S] clusters distributed among HoxE, HoxF and HoxU (Fig. 1). These cofactors mediate the electron transfer steps required for diaphorase activity with ferredoxin and NAD(P)H (16, 17, 18). Structural models of the HoxEFU complex show that HoxE forms a binding interaction with both HoxF and HoxU and that ferredoxin binding to HoxEFU includes a surface of HoxE, implicating a direct role for HoxE in supporting ferredoxin-dependent reactivity of HoxEFUYH (6, 7).

**Figure 1:**
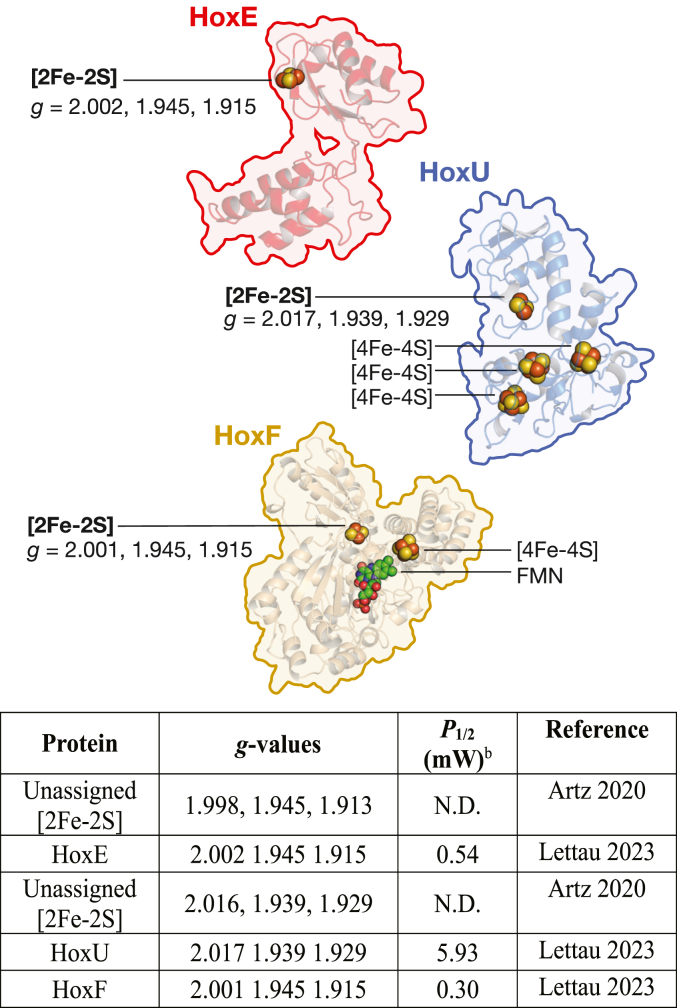
**Homology models of the individual HoxE, HoxF, and HoxU three-dimensional structures that comprise the HoxEFU diaphorase complex from *Synechocystis* PCC 6803** (7)**.** FeS cluster and flavin (FMN) cofactor composition depicted as spheres for the individual subunits (coloring: Fe, *dark red*; S, *yellow-orange*; C, *green*; N, *blue*; O, *red*). EPR signals and power to reach half-saturation (*P*_½_) values are indicated for the respective [2Fe-2S] clusters (*see*Table 1, N.D., not determined) (24).

HoxE shares sequence similarity with [2Fe-2S] binding proteins, including HydC of the electron bifurcating HydABC [FeFe]-hydrogenase (a HoxEFU homolog) in *Thermotoga maritima* (Fig. 1) and the NuoE subunit of the respiratory chain Complex I (19, 20, 21, 22, 23). Prior studies have identified EPR signals for the reduced [2Fe-2S] clusters of HoxEFU at *g* = 2.016, 1.939, 1.929 and *g* = 1.998, 1.945, 1.913 (Fig. 1). A later study found similar sets of *g*-values at *g* = 2.002, 1.945, 1.915; *g* = 2.001 1.945, 1.915; and *g* = 2.017, 1.939, 1.929 and specifically assigned them to HoxE, HoxF, and HoxU, respectively (7, 24). The reduction potentials of these clusters remained unidentified and functional understanding for how the clusters mediate electron transfer in HoxEFU in reactions with NAD(P)H and ferredoxin has not yet been determined. Supporting the essential role of the HoxE in the reactivity of HoxEFUYH, a deletion of HoxE resulted in loss of NAD(P)(H)-dependent H_2_ production (5, 15), and purified HoxFU lacking HoxE had attenuated rates of both NADH oxidation and ferredoxin-dependent NAD^+^ reduction (24). Additionally, the NAD(H)-dependent HoxFUYH [NiFe]-hydrogenases of *Hydrogenophilus thermoluteolus* (23), *Rhodococcus opacus* (25, 26) and *Ralstonia eutropha* (17, 27) that are unreactive with ferredoxin lack a HoxE subunit and are missing the [2Fe-2S] cluster in HoxF and a [4Fe-4S] cluster in HoxU. Thus, along with HoxE, there are additional iron-sulfur clusters in HoxF and HoxU that may be required to support reactivity of *S.* 6803 HoxEFUYH with ferredoxin.

In this study, we set out to solidify the understanding of the function of HoxE in mediating reactivity with ferredoxin by determining the magnetic properties and midpoint potentials for HoxE and the [2Fe-2S] clusters of HoxU and HoxF. A combination of EPR potentiometric titrations, SWV, and spectral simulation resolved the *E*_m_ value for each [2Fe-2S] cluster. By mapping this information onto a structural model of HoxEFU, important insights are gained to understanding the potential landscape for electron flow and the observed reactivities with diverse ferredoxins (7). We observed that the potential of purified HoxE, with *E*_m_ = −424 mV, shifted by more than 100 mV to < −525 mV when measured in the HoxEFU complex. The midpoint potentials of HoxF and HoxU were estimated at −419 mV and −349 mV, respectively. Together, the results demonstrated that the [2Fe-2S] midpoint potential of HoxE greatly depends on the structural and/or conformational context. These observations are used to propose a conformationally controlled, mechanistic framework for reactivity and electron transfer within the HoxEFU:ferredoxin complex.

## Results

### Spin quantification and magnetic properties of [2Fe-2S] clusters in purified HoxE and HoxU

**Figure 2:**
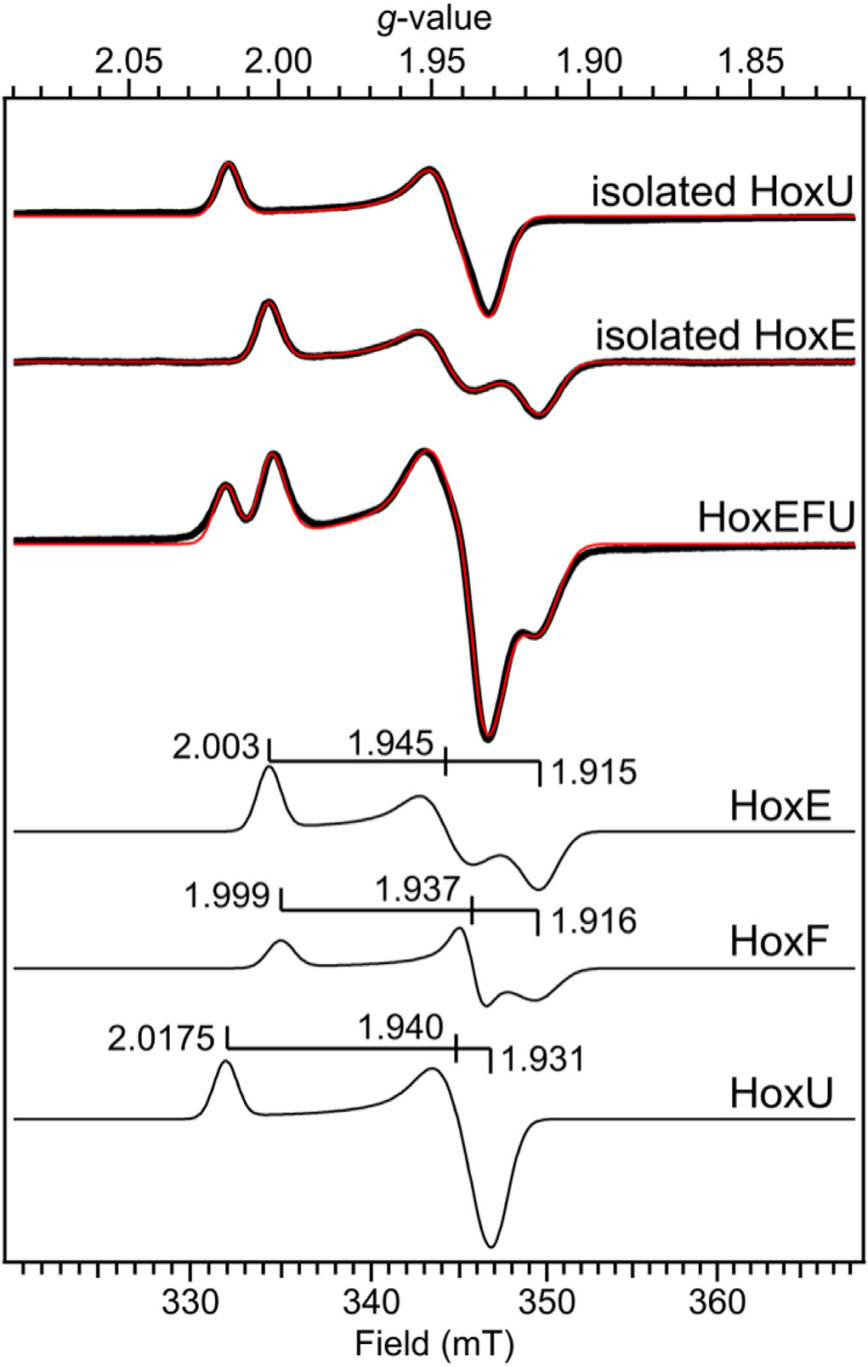
***Top*. EPR spectra (*black traces*) and simulations (*red traces*) ofpurifiedHoxE, HoxU, and HoxEFUreduced with 5 mM sodium dithionite, pH 8.3.***Bottom*. A breakdown of the HoxEFU simulation into the individual simulated [2Fe-2S] signatures for HoxE, HoxF, and HoxU is shown at the bottom with *g*-values indicated. See Table S1 description for more information on *g*_1_ value of HoxU. Data were collected at *T* = 40 K and *p* = 0.1 mW (HoxEFU, HoxE) or *p* = 1 mW (HoxU).

Past investigation of *Synechocystis* HoxE revealed a characteristic [2Fe-2S] cluster binding motif, with homologs to HoxE (HydC in *T. maritima* and NuoE in *Thermus thermophilus*) also observed to bind [2Fe-2S] clusters (20, 21, 22). HoxE protein was purified to homogeneity and contained 1.2 Fe atoms/mol (Fig. S1, Table S2). After reduction with sodium dithionite (NaDT), the EPR spectrum displayed a rhombic signal at *g* = 2.003, 1.945, 1.915 (Fig. 2, Table S1). Simulation of the signal verified the *g*-value assignments and resulted in an excellent fit to the experimental data (Fig. 2, Tables 1 and S1). Overall, the similarity of the signal to the EPR signals previously characterized from HydC and NuoE support a [2Fe-2S] cluster assignment (Table 1). Variable-power EPR data of HoxE collected at 40 K (Fig. S2) produced a characteristic power-saturation curve with saturation of the signal beginning at powers above 0.1 mW and a power to reach half saturation (*P*_1/2_) value of 5.0 ± 0.4 mW at 40 K and a *b* value of 1.3 ± 0.07, indicative of inhomogeneous broadening (Fig. S2, Table 1) (28). Variable-temperature EPR data collected on HoxE at a power of 0.1 mW showed a maximal signal intensity near 40 K (Figs. 2 and S2). The signal could be observed up to at least 70 K with minimal temperature broadening. These results are in agreement with previous EPR studies and are consistent with the presence of a redox active [2Fe-2S]^2+/1^ cluster in HoxE, which typically relax slower than *e.g.*, [4Fe-4S] clusters and necessitate higher temperatures and/or lower powers to observe without signal saturation (29, 30).

**Table 1:** Properties of the [2Fe-2S] clusters in HoxEFU and related complexes

Protein^a^	Form	*g*-values	T_opt_ (K)^b^	*P*_1/2_ (mW)^c^	*E*_m_ (mV)^d^	Reference
TtNuoE	Isolated	1.996, 1.94, 1.920	40	N.R.	−295	Birrell 2013
DfHndA	Isolated	2.000, 1.950, 1.915	100	N.R.	−395	De Luca 1998
TmHydC	isolated	2.005, 1.950, 1.919	40	N.R.	−361	Birrell 2016
TmHydC	Isolated	2.000, 1.949, 1.914	55	N.R.	−352	Verhagen 2001
HoxE	Isolated	2.002, 1.945, 1.915	N.R.	0.54	N.R.	Lettau 2023
HoxE	Isolated	2.003, 1.945, 1.915	40	5.0 ± 0.4	−424	This work
HoxE	HoxEFU	2.002, 1.945, 1.915	N.R.	0.45	N.R.	Lettau 2023
HoxE	HoxEFU	2.003, 1.945, 1.915	U	U	< −525	This work
HoxU	HoxEFU	2.017, 1.939, 1.929	N.R.	0.01–5.93	N.R.	Lettau 2023
HoxU	HoxEFU	2.017, 1.940, 1.931	30	8.1 ± 2.8	−349 ± 7	This work
HoxU	Isolated	2.017, 1.944, 1.931	40	14.6 ± 3.7	N.R.	This work
HoxF	HoxFU	2.001, 1.945, 1.915	N.R.	0.30	N.R.	Lettau 2023
HoxF	HoxEFU	1.999, 1.937, 1.916	U	U	−419 ± 2	This work

N.R., not reported; U, unresolved.

a Proteins isolated from *Thermus thermophilus* (Tt), *Desulfovibrio fructosovorans* (Df), *Thermotoga maritima* (Tm).

b The optimal temperature (T_opt_) or temperature range of the signal without saturation. Determined either for clusters in isolated proteins (isolated), or in the functional complex (complex) from fits to temperature saturation curves, Figs. S2 (HoxE), S3 (HoxU) and S5 (HoxEFU).

c Microwave power where the signal intensity is half-saturated from the fits to power saturation curves, Figs. S2 (HoxE), S3 (HoxU) and S5 (HoxEFU). *P*_1/2_ values were determined by fitting power curves as described in the Experimental procedures section. The corresponding *b* values were 1.3 ± 0.07 for isolated HoxE, 0.39 ± 0.07 for isolated HoxU, and 0.35 ± 0.09 for HoxU in HoxEFU.

d Determined by potentiometric EPR (TtNuoE (21), TmHydC (20), or HoxEFU complex, this study) or square wave voltammetry (TmHydC (20), isolated HoxE, this study). Standard deviations from Nernst fits are given for *E*_m_ values of HoxF and HoxU (Fig. S7).

The HoxU subunit was also purified to homogeneity and contained 11 Fe-atoms/mol (Fig. S1, Table S2) and displayed a rhombic EPR signal at *g* = 2.0165, 1.944, and 1.931 (Figs. 2 and S3). Variable-temperature EPR data on HoxU was collected at 1 mW and displayed a maximal signal intensity at 40 K, while variable-power data collected at 40 K revealed power saturation of the signal above 1 mW with a *P*_1/2_ = 14.6 ± 3.7 mW (Fig. S3, Table 1). Our new results on the properties of this FeS cluster in HoxU are consistent with the assignment to a redox active [2Fe-2S]^2+/1^ cluster (7, 24).

### Resolving the [2Fe-2S] cluster EPR signals of HoxEFU

The fully assembled HoxEFU complex contains seven FeS clusters in total, comprised of four [4Fe-4S] clusters and three [2Fe-2S] clusters, one of which is in the HoxE subunit. Having determined the EPR signal properties of the [2Fe-2S] cluster in HoxE, the EPR spectra of the purified HoxEFU complex were analyzed to determine the magnetic properties of the remaining [2Fe-2S] clusters. HoxEFU was purified and determined to have 20 ± 2 Fe-atoms/mol and 0.98 ± 0.04 FMN/mol (Table S2). Variable temperature and power EPR spectra of NaDT reduced HoxEFU (Fig. S4) showed complex signals from multiple reduced FeS clusters. A spin-quantification of the HoxEFU spectrum collected at 1 mW and 15 K, which allows for the detection of all the FeS clusters, gave a value of 7.3 spins/mol (Table S2). This compares well to the expected seven spins/mol from 3x[2Fe-2S] and 4x[4Fe-4S] clusters/mol HoxEFU. To analyze the presence of an FMN radical, a sample of HoxEFU was reduced with 10 mM NADH, and an EPR spectrum collected at *T* = 200 K, *p* = 10 mW. The spectrum revealed an isotropic-type signal centered at *g* ∼ 2 consistent with an FMN radical (Fig. S4).

The reduced [4Fe-4S] clusters of HoxEFU give rise to strong, overlapping EPR signals at lower temperatures (*e.g.* <25 K); therefore, further EPR analysis of [2Fe-2S] cluster signals was performed at 40 K. This temperature is ideal for observing the [2Fe-2S] clusters in HoxE and HoxU and minimizes [4Fe-4S] cluster contributions to simplify the spectral analysis. Simulation of the NaDT reduced HoxEFU spectrum revealed signals at *g* = 2.003, 1.945, 1.915 and *g* = 2.0175 1.940 1.931, which are consistent with signals of purified HoxE and HoxU, respectively (Fig. 2, Tables 1 and S1). An additional component was observed in the NaDT reduced HoxEFU spectra and was resolved by simulation at *g* = 1.999 1.937 1.916 (Tables 1 and S1), which we assign to the [2Fe-2S] cluster signal of HoxF. Collectively, the EPR signals align with our previously determined [2Fe-2S] cluster type signals at *g* = 2.016, 1.939, 1.929, and *g* = 1.998, 1.945, 1.913 (7). These new HoxE, HoxF, and HoxU signal assignments are similar to the signals reported in a prior study with *g* = 2.002, 1.945, 1.915; *g* = 2.001 1.945, 1.915; and *g* = 2.017, 1.939, 1.929 and that were assigned to HoxE, HoxF, and HoxU, respectively (24). The power and temperature saturation behavior of the [2Fe-2S] cluster signals in the EPR spectrum of NaDT reduced HoxEFU were also determined for comparison to that of the reduced [2Fe-2S] clusters in purified HoxE and HoxU (Figs. S4 and S5). Due to the spectral overlap of HoxE and HoxF [2Fe-2S] cluster signals, the *P*_1/2_ values could not be assessed individually. However, a fit of the signal at *g* ∼ 2.002, which includes contributions from both HoxE and HoxF, gave a *P*_1/2_ value of 8.3 ± 1.0 mW at 40 K (Fig. S5, Table 1). Analysis of the HoxU [2Fe-2S] cluster signal in the HoxEFU complex gave a *P*_1/2_ = 8.1 ± 2.8 mW at 40 K, with the maximal signal intensity prior temperature saturation (T_opt_) obtained at 30 K (Fig. S5, Table 1), compared to values of *P*_1/2_ = 14.6 ± 3.7 mW and T_opt_ = 40 K for purified HoxU. The differences in the power and temperature saturation behavior of the [2Fe-2S] cluster signal from purified HoxU *versus* in the HoxEFU complex are likely due to perturbations of the magnetic interactions, for example, dipolar couplings, between this [2Fe-2S] cluster and the other reduced FeS clusters. This is further supported by the *b* values of 0.39 ± 0.07 (Fig. S3, HoxU purified) and 0.35 ± 0.09 (Fig. S5, HoxU in HoxEFU) we obtained from fitting of the HoxU [2Fe-2S] cluster power data, which indicate the influence of such interactions in both cases (see Experimental procedures) (28).

### The reduction potentials of the HoxEFU [2Fe-2S] clusters

**Figure 3:**
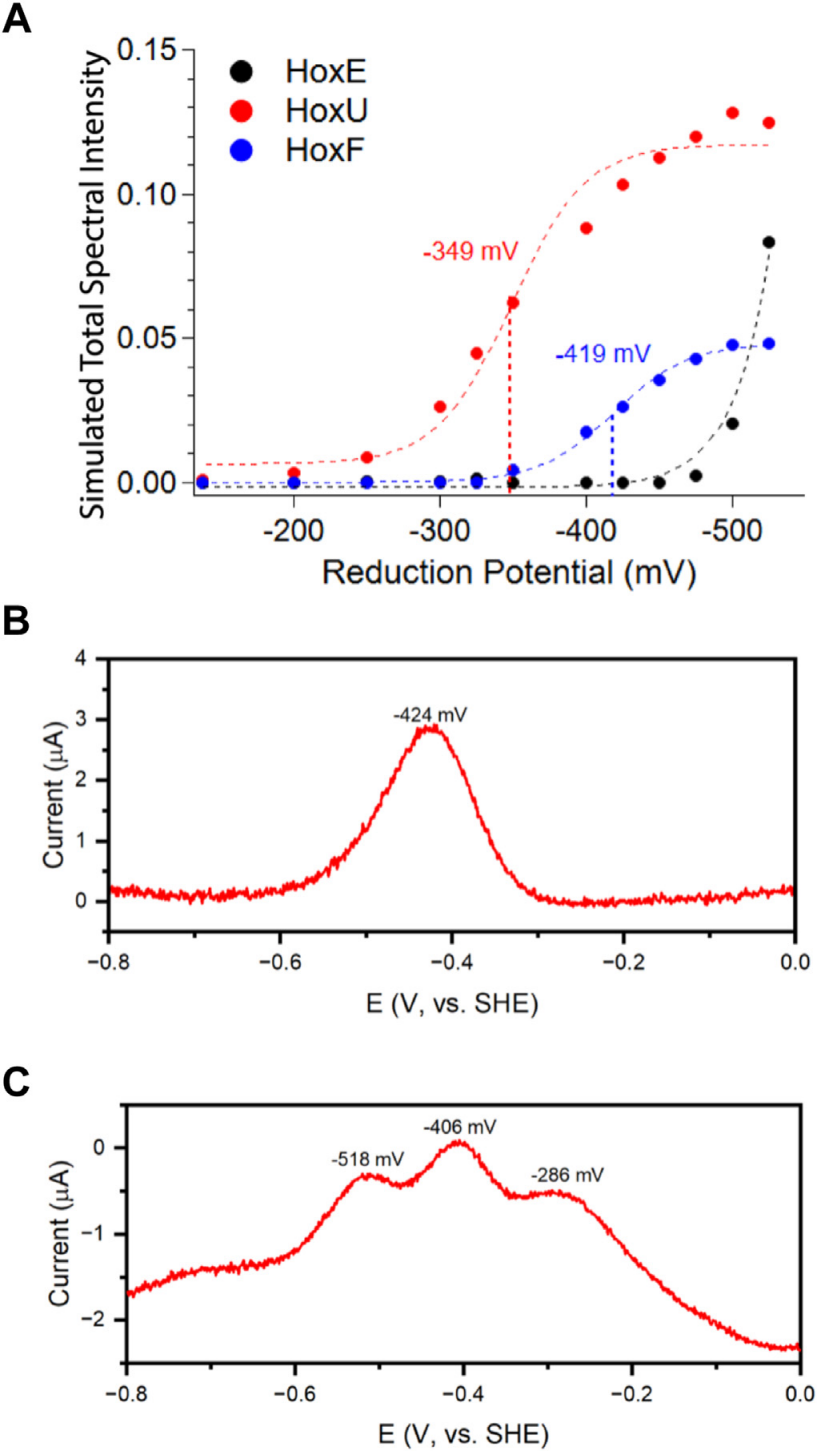
**EPR potentiometric titrations of HoxEFU and square wave voltammetry (SWV) measurements of HoxE and HoxEFU.***A*, the simulated total signal intensity for each of the HoxEFU [2Fe-2S] clusters (*dots*) plotted *versus* potential and shown fit to the one-electron Nernst equation (*dashes*). Total intensities obtained from the simulation of potentiometric EPR data are shown in Fig. S6 (data collected at *T* = 40 K, *p* = 0.1 mW). For HoxE data, a fit is shown to a Nernstian curve with *E*_m_ = −550 mV. *B*, SWV of purified HoxE (50 μM concentration) performed in the reducing direction using a PGE electrode. The peak at *E* = −424 mV is assigned to the reduction of the [2Fe-2S] cluster. *C*, SWV of purified HoxEFU (50 μM concentration) was performed in the reducing direction using a PGE electrode.

To measure the midpoint potential (*E*_m_) of the FeS clusters of HoxE and HoxEFU, we used potentiometric EPR (Fig. 3 and S6) and SWV (Figs. 3 and S8) measurements performed on purified HoxE and HoxEFU. For purified HoxE, the SWV revealed a single peak at −424 mV *versus* the standard hydrogen electrode (SHE) and is consistent with an *E*_m_ ∼ −424 mV for the [2Fe-2S] cluster. For the EPR redox titration, HoxEFU was equilibrated with redox dyes and titrated with NaDT over a potential range of −138 mV to −525 mV following a previously established protocol (31). Due to the overlapping EPR signals of the HoxE and HoxF [2Fe-2S] clusters (Fig. S4), it was not possible to determine the *E*_m_ values from raw signal intensities in the potentiometric EPR spectra. Therefore, spectral simulations were performed and resulted in high-quality fits of the spectra (Fig. S6). At potentials positive of −375 mV, the HoxU signal in the HoxEFU redox titration is nearly identical to that of the purified HoxU, the exception being a upfield shift in *g_2_* value from 1.944 (purified HoxU) to 1.940 (HoxEFU) (Figs. 2 and S6, Table S1). However, the *g*_1_ value of the HoxU signal shifts as the reduced iron-sulfur cluster content increases. At potentials between −375 and −475 mV, the *g*_1_ value shifts downfield toward a final value of 2.0175, concomitant with the reduction of the [2Fe-2S] cluster of HoxF. Notably, the downfield shift stops before the [2Fe-2S] cluster of HoxE starts to contribute at the lowest potentials.

The simulated total spectral intensities of each [2Fe-2S] cluster species were plotted *versus* potential (Figs. 3 and S7). Fits of the simulated intensities of the [2Fe-2S] clusters of HoxU and HoxF to construct Nernst curves resulted in *E*_m_ values of −349 mV and −419 mV, respectively. It was not possible to fit the HoxE potentiometric data to a Nernst equation due to there being too few data points and we estimate the *E*_m_ value of the [2Fe-2S] cluster of HoxE is < −525 mV *versus* SHE in the HoxEFU complex (Figs. 3 and S7, Table 1). For SWV, when scans were taken in the reducing direction, three broad peaks were observed that were centered at −518 mV, −406 mV, and −286 mV *versus* SHE (Fig. 3), whereas two weaker peaks at −521 mV and −384 mV were observed in the oxidizing direction (Fig. S8). The observation of several broad, overlapping peaks agrees with the presence of multiple iron-sulfur cofactors with similar *E*_m_ values. Differences in the compositions and intensities of redox peaks are likely to result from differences in the films or possibly from differences in chemical steps associated with the reduction-oxidation processes (32). We also note the presence of a weak feature at ∼ −110 mV that may be accounted for as free FMN (*E*_m_ of the semiquinone to hydroquinone ∼ −100 mV) (33).

While definitive cluster assignments cannot be made from this SWV data alone, the general location of the peaks can be used to support the midpoint potentials determined by EPR analysis. The peak at −518 mV is consistent with the lower *E*_m_ value for HoxE assigned from the potentiometric EPR analysis of HoxEFU. The large signal at ∼ −406 mV supports assignment to a combination of HoxU and HoxF clusters, where the broader peak can be attributed to multiple iron-sulfur cluster contributions. The peak at −286 mV likely is due to the reduction of the oxidized HoxF FMN. Collectively, the SWV and potentiometric EPR results are consistent with the *E*_m_ values assigned to each of the three [2Fe-2S] clusters spanning a range of ∼190 mV. Strikingly, the *E*_m_ of purified HoxE is shifted from −424 mV to < -525 mV, or >100 mV, when associated with the HoxEFU complex.

### A potentiometric/conformational model for reactivity of HoxEFU with various ferredoxins

The HoxEFU:ferredoxin binding model previously proposed from mass spectrometry crosslinking data (7) suggests that HoxE may be involved in directing electron transfer with ferredoxin. The arrangement of subunits and [2Fe-2S] clusters in the structural model together with the information gained here can be used to begin to establish a structural and thermodynamic rationale for ferredoxin reactivity that has been observed. The direction and rate of the diaphorase reaction catalyzed by HoxEFU as described by Equation 1.

(1) $$\text{Fd}x_{\text{Red}}+\text{NAD}{\left(P\right)}^{+}\;⇄\;\text{Fd}x_{\text{Ox}}+\text{NAD}\left(P\right)H$$

is influenced by the relative *E*_m_ of the Fdx *versus* the NAD(P)H/NAD(P)^+^ couple. For example, HoxEFU catalyzes NADH production, but not NADH oxidation, in the presence of reduced Fdx1, Fdx4, or Fdx11 (7). These Fdx’s have *E*_m_ values in the range of −412 to −460 mV (Figs. S9 and S10) (34, 35), which are all more negative than *E*_m_ ∼ −320 mV for NAD(P)^+^/NAD(P)H. The highest rate of NADH production (Table 2) was measured with reduced Fdx4, *E*_m_ = −460 mV. Likewise, Fdx2, which has an *E*_m_ = −246 mV, was the only Fdx able to drive NADH oxidation.

**Table 2:** HoxEFU diaphorase activity and physical properties of various ferredoxins

Fdx	*E*_m_ (*versus* SHE)^a^	Δ*E*_HoxE(FU)/Fdx_^b^	Δ*E*_HoxE/Fdx_^b^	Δ*E*_HoxF/Fdx_^b^	*k*_obs_ NAD^+^ reduction (min^−1^)^c^	*k*_obs_ NADH oxidation (min^−1^)^c^
Fdx1	−412	−113	−12	−7	0.63	N.A.
Fdx4	−460	−65	+36	+41	0.81	N.A.
Fdx11	−416	−111	−8	−3	0.10	N.D.
Fdx2	−246	−279	−178	−173	N.A.	0.02

a Fdx4 and Fdx11, this study, Figs. S9 and S10, respectively; values for Fdx1 and Fdx2 obtained from refs. 35 and 36, respectively.

b Δ*E*_HoxE(FU)/Fdx_ = *E*_mHoxE(FU)_−*E*_mFdx_, where *E*_mHoxE(FU)_ is *E*_m_ = −525 mV *versus* SHE, or the value of HoxE in HoxEFU. Δ*E*_HoxE/Fdx_ = *E*_mHoxE_−*E*_mFdx_, where *E*_mHoxE_ = −424 mV *versus* SHE. Δ*E*_HoxF/Fdx_ = *E*_mHoxF_−*E*_mFdx_, where *E*_mHoxF_ = −419 mV *versus* SHE. See Experimental procedures.

c Values from Artz *et al.* (7), N.A. = no activity was observed, N.D. = not determined. NAD(P)^+^/NAD(P)H, *E*_m_ = −320 mV.

An interpretation based on the outcome of this work is that HoxE adopts two structural conformations in the HoxEFU complex, which are in equilibrium. Under this assumption, the directionality of the reaction catalyzed in Equation 1 can be interpreted as being controlled by conformational changes in HoxE that affect the [2Fe-2S] cluster potential. For example, electron transfer from Fdx4 (−460 mV) to HoxEFU is favorable if HoxE is in the high potential conformation, with an *E*_m_ of −424 mV (Δ*G*_ET_ = −36 mV) *versus* < −525 mV (Δ*G*_ET_ > +65 mV), which agrees with the measured results. Reactions with Fdx2 also have overall favorable energetics when HoxE is in the high potential conformation with a Δ*G*_ET_ = −178 mV (Table 2) (35). A switch from the high potential to the low potential regime of HoxEFU would then favor a subsequent reduction of NAD(P)^+^. This model is consistent with the low *k*_obs_ for NADH production with Fdx2 (Table 2).

## Discussion

While the diaphorase activity of HoxEFU has been investigated, the specific details of this process, beginning with electron flow between ferredoxin and pyridine, remain unknown, as do the roles of the HoxEFU cofactors. Structural work by Feng *et al.* demonstrated a necessity for conformational changes by the homologous HydABC complex during electron transfer (36). This, combined with studies by Birrell *et al.* that illustrated the impact on redox potential of changing the environment around a cluster, suggests variability in the *E*_m_ of iron-sulfur clusters in these complexes (22). Understanding the HoxEFU diaphorase complex may offer better insight into the key factors differentiating HoxEFUYH from related [NiFe]-hydrogenases that demonstrate more readily reversible diaphorase activity, which requires the breakdown of HoxEFU into the individual proteins, starting with HoxE (37, 38, 39).

The EPR of HoxE alone confirmed the proposal that the cluster is a [2Fe-2S] cluster based on the observed signal at *g* = 2.003, 1.945, 1.915 and its relatively slow relaxation as assessed by temperature-dependent measurements. For HoxE alone, the *E*_m_ of the [2Fe-2S] cluster was determined by SWV to be approximately −424 mV, which is about 65 mV lower than those reported for the HydC (−352 to −361 mV) and NuoE (−370 mV) homologs (20, 22, 40). For the HoxEFU complex, complimentary EPR potentiometric and SWV measurements show that the [2Fe-2S] cluster *E*_m_ is shifted significantly negative to below −525 mV. The potential differs from the −419 mV potential of the [2Fe-2S] cluster in HoxF and the −349 mV potential of the [2Fe-2S] HoxU cluster, supporting the conclusion that the environment surrounding FeS clusters in HoxEFU has a significant impact on the midpoint potential. The observation that the *g*-values of the [2Fe-2S] cluster signal are the same in HoxE and the HoxEFU complex suggests that there are no major changes to the [2Fe-2S] cluster structure that might influence the *E*_m_. Other factors, such as changes in the solvation environment due to binding interactions have been found to influence the *E*_m_ of another [2Fe-2S] protein (41, 42). For example, this effect has been reported as the influence of the 2x[4Fe-4S] cluster subunit, PsaC, on the *E*_m_ of the F_x_ [4Fe-4S] cluster in Photosystem I. In the absence of PsaC which binds to the surface adjacent to F_x_, there is a positive shift in the *E*_m_ of F_x_ (43), which is similar in magnitude to what is observed for the shift in the *E*_m_ of HoxE in the absence of HoxFU. In the context of a HoxEFU structure, HoxE might also undergo changes in conformation. In support of this hypothesis, the HoxE homolog, HydC of the bifurcating *A. mobile* HydABCSL [NiFe]-hydrogenase complex, is proposed to adopt two conformations, one mediating electron transfer with ferredoxin and the other with FMN of HydB (36). HoxE may function by a similar mechanism to coordinate and control electron flow within HoxEFU and involve a modulation of the *E*_m_ of the [2Fe-2S] cluster. Rather than catalyzing the strict coupling of three half-reactions, which defines electron bifurcation, a conformation-induced change in *E*_m_ of HoxE may control the reaction kinetics and directionality of electron flow between pairs of substrates, *i.e.*, ferredoxin and NAD(P)H.

An alternative explanation for the downshift in *E*_m_ of HoxE in the HoxEFU complex is the effect of anti-cooperativity from nearby reduced clusters in either HoxF or HoxU. In multi-cluster systems, this effect is known to result in a negative shift in the *E*_m_ of a cluster when nearby clusters are reduced. An example of this effect has been demonstrated for the reduction potentials of 2x[4Fe-4S] cluster ferredoxin of *Clostridium pasteurianum* and for iron-sulfur clusters in nitrate reductase (44). For *C. pasteurianum* ferredoxin, although the two clusters were found to share the same *E*_m_ value, reduction of both clusters required a more negative potential than for reduction of only one cluster (45).

Overall, the outcome of this study assigned the *E*_m_ values for each of the three [2Fe-2S] clusters of HoxE, HoxF, and HoxU. In the context of the proposed structural models for the HoxEFU:ferredoxin binding complex (7, 24), these results provide insights into the thermodynamic landscape of electron flow to explain the variations in NAD(P)H:ferredoxin reactivity with ferredoxins having different *E*_m_ values (Table 2). The [2Fe-2S] clusters of HoxE and HoxF are common to HoxEFUYH complexes that react with ferredoxin and are not present in HoxFUYH complexes that do not react with ferredoxin (17, 23, 25, 26, 27). The *E*_m_ value of HoxE in the high potential state, or HoxF, is similar to the *E*_m_ value of Fdx1, Fdx4, and Fdx11 (7, 46) for favorable electron transfer into HoxEFU according to the measured reactivity (Table 2). Whereas the electron injection from the more positive Fdx2 is disfavored. A shift of HoxE into a low *E*_m_ conformation may function to gate the energetics of electron transfer within HoxEFU dependent on the binding of pyridine nucleotide and ferredoxin substrates. Future investigations will aim to fully decipher the functions of HoxE, HoxF and HoxU in managing electron flow within HoxEFU complex and in defining the diaphorase reactivity.

## Experimental procedures

### Gene construction

The gene encoding HoxE from *Synechocystis* PCC 6803 was codon optimized and subcloned into the carbenicillin-resistant pET-21b vector (Novagen) by GenScript. A Strep-II tag was added to the N-terminus of HoxE (7). The gene encoding HoxU in *Synechocystis* PCC 6803 was also codon optimized and subcloned into the pET-21b plasmid. An N-terminal Strep-II tag was added to the HoxU gene. The expression construct used for HoxEFU was described previously (7).

### Expression and purification of HoxE, HoxU, HoxEFU, Fdx4 and Fdx11

Fdx4 and Fdx11 were purified as previously described (7). Expression and purification of HoxEFU was carried out following previously described protocols, with a few changes (7). The HoxEFU expression plasmid was transformed into electrocompetent BL21(DE3)Δ*iscR* kanamycin (Kn) resistant cells and plated on Luria-Bertani (LB) agar plates supplemented with Kn (50 mg/L) and carbenicillin (Carb, 100 mg/L). A single colony was used to inoculate a 100 ml overnight starter of Terrific Broth (TB) supplemented with Kn (50 mg/L) and Carb (100 mg/L), and the culture was grown overnight at 37 °C with 250 RPM shaking. The following day, a 1:100 dilution of the culture was used to inoculate 8 L of TB media supplemented with Kn (50 mg/L) and Carb (100 mg/L). Upon reaching an OD_600_ of 0.5 to 0.7, the cells were induced with 500 μM IPTG and 1 mM lactose and ferric ammonium citrate (4 mM final) and FMN (10 μM final) were added. The cells were continued to shake at 37 °C for 1 h, prior to being mixed with 0.5% glucose and 10 mM sodium fumarate dibasic. The cells sat for an additional 30 to 40 min at room temperature before the addition of cysteine (2 mM final), with ensuing sparging by argon gas for 16 h. The remaining protocol was carried out under strict anaerobic conditions. The cells were harvested *via* centrifugation at 4 °C and 6600*g*. Pelleted cells were resuspended inside a Coy chamber in 50 mM Tris, 300 mM NaCl, 5% glycerol, 2 mM dithionite, pH = 8.3 buffer. Resuspensions were transferred to a septum-sealed vial and frozen and stored at −80 °C.

For purification of HoxEFU, the cells were thawed, treated with EDTA-free protease inhibitor tablets, DNase I, and lysozyme, followed by ∼10 passes through a microfluidizer located inside a Coy chamber. Lysed cells were centrifuged for 1 h at 149,000*g*, with the resulting supernatant loaded onto a high-capacity Strep-Tactin XT-4Flow resin (IBA Life Sciences, Germany) located inside an MBraun glove box. After a wash phase with 50 mM Tris buffer, 300 mM NaCl, 5% glycerol, and pH = 8.3, the purified protein was eluted with the same buffer containing 25 mM biotin. The yields of HoxEFU were ∼3 mg/L. Iron and flavin totals were verified as previously described (47, 48) with values in Table S2.

Expression and purification of HoxE and HoxU was performed following a similar protocol to HoxEFU with several changes. The HoxE expression plasmid was transformed into Bl21(DE3) cells, and all media was TB supplemented with Carb (100 mg/L). No FMN was added, and ferric ammonium citrate was added to 2.5 mM final concentration, and cysteine was added to 1 mM concentration. The remainder of the purification protocol for HoxE was the same as for HoxEFU and purified protein yields were about 0.5 mg/L. The expression and purification protocol for HoxU was identical to that of HoxEFU with the exception that FMN was not added. HoxE and HoxU were verified to be individually purified *via* SDS-PAGE gel electrophoresis (Fig. S1) with iron totals verified using the same method as HoxEFU.

### Potentiometric EPR redox titrations and measurements

EPR-monitored redox titrations were performed following the protocol from Mulder *et al.* with samples entirely prepared under strict anaerobic conditions in an MBraun glove box maintained under N_2_ atmosphere (31). A 50 μM sample of HoxEFU was prepared in 50 mM Tris, 300 mM NaCl, 5% glycerol, and loaded into a custom glass cell (Allen Scientific Glass, Boulder, CO) with a conical bottom and ports for both sample removal and measurement of the solution potential by a tri-electrode (Thermoscientific Orion 9678BNWP). A redox dye mixture was added to the sample with final concentrations of 6 μM indigo carmine, 6 μM phenosafranin, 6 μM benzyl viologen, 6 μM methyl viologen, and 6 μM ethyl viologen. The protein was thoroughly equilibrated with the dye mixture by constant stirring and monitoring of the solution potential until the reading did not change. After equilibration, NaDT was added (concentrations varied) to reach the desired potentials. After equilibrium of the sample at desired potentials, ∼150 to 170 μl were removed and added to an EPR tube (Wilmad). The EPR sample was then capped with a septa, removed from the glove box, and immediately frozen and stored in liquid nitrogen.

### EPR data collection and processing

Continuous-wave X-band EPR measurements were made using a Bruker Elexsys E−500 spectrometer equipped with a super high-Q resonator (Bruker), cryogen-free helium cryostat (ColdEdge Technologies), and MercuryITC temperature controller (Oxford Instruments). All spectra were collected at a frequency of 9.38 GHz, using a modulation frequency of 100 kHz and a modulation amplitude of 10 G. Specific powers and collection temperatures were noted in figure legends.

Data were baseline corrected either through subtraction of a polynomial function in IgorPro v.9 (https://www.wavemetrics.com/software/igor-pro-9) or by taking an average of the first and last 50 points of the spectra and subtracting a value from each point based on a linear gradient between the initial and final average.

### Analysis of variable power and temperature EPR data

Variable-power data (Figs. S2–S4) were obtained from the scaling of raw EPR signal intensities (*S*) according to the relationship *S* ∝ √*P* for each spectrum (29). Variable-temperature EPR data (Figs. S2–S4) were corrected for the Curie Law through the multiplication of the spectra by their respective collection temperatures. Temperature- and power-dependent saturation behavior was assessed from these normalized spectra by monitoring intensity changes at g-values characteristic of the individual cluster’s signal and plotting against either the collection temperature or power, respectively (Figs. S2–S4). To obtain a P_1/2_ value, the power-dependent data were fit to Equation 2:

(2) $$\frac{s}{\sqrt{P}}=\frac{1}{{\left(1+\frac{P}{P_{1/2}}\right)}^{1/2}}$$

Where *S* is the intensity of the EPR signal, *P* is the microwave power, and *b* is a parameter representing the origin of the fit line broadening which varies between one (inhomogeneous broadening) and 3 (homogenous broadening) (29). A *b* value less than one indicates the additional influence of dipolar interactions on the signal saturation behavior, *e.g.*, from spin-coupling to nearby paramagnetic centers (28).

### Spin quantification and simulation of EPR data

EPR simulations were performed on non-normalized data using the Easyspin toolbox version 6.01 (49) within Matlab v.2024a (Mathworks, https://www.mathworks.com). The exact simulation parameters are given in Table S1. For potentiometric data fitting, a radical signal at *g* = 2.003 (linewidth = 1.8 mT) was included to account for contributions from the redox dye mediator cocktail, with this species accounting for <1% of the total simulated intensity in all samples. Spin quantification was performed by comparing the double integral of the sample spectra to that of a copper sulfate standard (75–100 uM CuSO_4_ in triethanolamine) collected at the same temperature under non-saturating conditions.

### Determination of E_m_ potentials from EPR redox titration data

To estimate the *E*_m_ values for each cluster, each potentiometric EPR spectrum (Fig. S6) was simulated using the identified signals for the three [2Fe-2S] clusters as determined from the fully reduced (+NaDT) data (Fig. 2, Table S1). To avoid misrepresentation of signal contribution due to saturation, the entire redox titration was collected at 40K and 0.1 mW (Table 2). By fitting each simulation to the raw data intensity (after correction for sample dilution), the simulated intensity contributions for each cluster can be plotted *versus* sample potential to generate potentiometric data for analysis using the Nernst equation (Fig. 3). These data were then fit to the Nernst equation (Equation 3) using a custom fit function in IgorPro v9, with the 95% confidence bands for each fit also shown (Fig. S7).

(3) $$f\left(E\right)=\frac{Simulated\;cluster\;intensity}{1+e^{\left(E-E_{m}\right)\frac{F}{RT}}}$$

Variables: *E* = potential, *E*_m_ = midpoint potential, *F* = Faraday constant = 96,480 C/mol, *R* = gas constant = 8.314 J/K∗mol, *T* = temperature in Kelvin.

### Protein-film square wave voltammetry

Protein-film square wave voltammetry (SWV) was performed in an MBraun anaerobic chamber following the protocol by Wise *et al.*, with several changes (48). For HoxE, HoxEFU, and Fdx11, a three-electrode, 100 ml standard cell (Pine Research, RRPG021) using a fixed disk pyrolytic graphite edge (PGE) working electrode (Pine Research, AFE1E0505GE), a low-profile Ag/AgCl/saturated KCl reference electrode (Pine Research, RRPEAGCL), and platinum wire counter electrode (BASi, MW_4130) were used. Using alumina slurry (BASi, CF-1050) and alumina polishing pads (BASi, MF-1040), the electrode was polished and rinsed with water. The protein being tested was then applied to a working electrode with at least 10 min for incubation before being placed in the cell containing 150 mM HEPES, 200 mM NaCl, 5% glycerol, pH = 8.3. Between measurements, the protein was reapplied due to loss of film. Data was collected with potential applied in oxidizing (negative-to-positive) and reducing (positive-to-negative) directions with time increments of 0.001 V, amplitude of 0.025 V, frequency of 10 Hz, and sensitivity of 1 × 10^−6^ A × V^−1^ with a CH Instruments 630C Potentiostat and CHI630C software. Simultaneous sampling of both current and potential at a rate of 1 MHz (10^6^ s^−1^) for each channel was used for data acquisition. The buffer background was taken by using the same conditions with the protein biofilm omitted. Following conversion from the Ag/AgCl/saturated KCl reference electrode by addition of 199 mV, all values for potential are reported *versus* SHE. For Fdx4, a gold working electrode (BASi, MF-2014) was used with 3uL of Fdx4 protein applied. The remainder of the protocol, including the reference and counter electrodes used, remained unchanged from the protocol discussed above (7, 31, 47, 48, 50).

## Data availability

All data are contained within the manuscript and supporting information.

## Supporting information

This article contains supporting information (47, 48).

## Conflict of interest

The authors declare that they have no conflicts of interest with the contents of this article.
