# Supplementary material for: Functional roles of the [2Fe-2S] clusters in Synechocystis PCC 6803 Hox [NiFe]-hydrogenase reactivity with ferredoxins
Source: J Biol Chem. 2024 Oct 28;300(12):107936. doi: 10.1016/j.jbc.2024.107936 (PMC11647496; doi:10.1016/j.jbc.2024.107936)
Supplement: Supporting information [file mmc1.docx]

Supporting Information

Functional roles of the [2Fe-2S] clusters in *Synechocystis* PCC 6803 Hox [NiFe]-hydrogenase reactivity with ferredoxins

**Matthew R. Blahut, Michael E. Dawson,^a^ Effie C. Kisgeropoulos,^a^ Anastasia Ledinina, David W. Mulder, Paul W. King***

Biosciences Center, National Renewable Energy Lab, Golden CO, 80401

Running title: *Roles of HoxEFU [2Fe-2S] clusters*

*Corresponding author, [paul.king@nrel.gov](mailto:paul.king@nrel.gov)

^a^These authors made equal contributions to the work.

**Supplemental Figures**


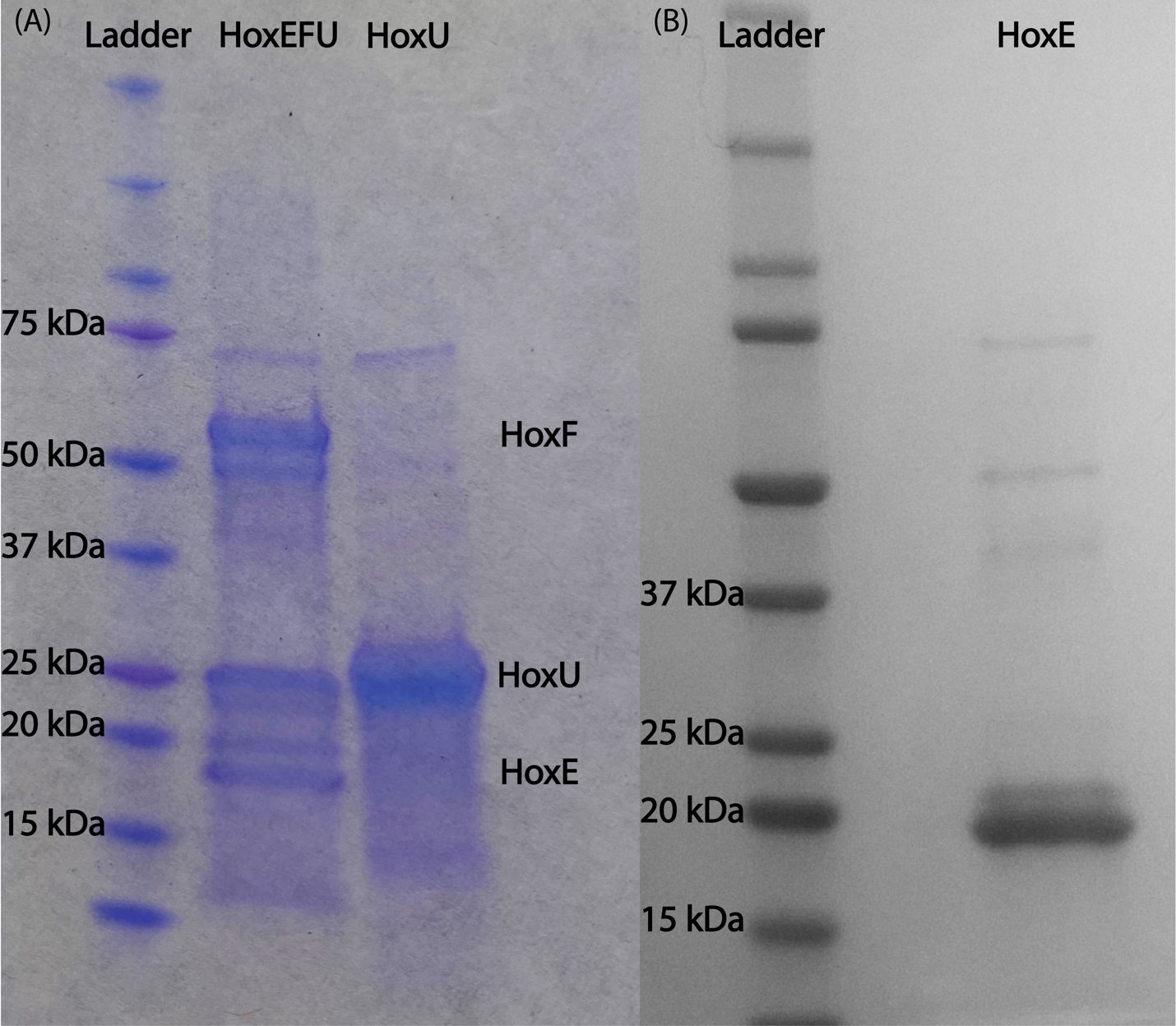


**Figure S1**. SDS-PAGE of purified (A) HoxEFU and HoxU, and (B) purified HoxE. (A). 8 µg of HoxEFU and HoxU (B) 4 μg HoxE. Expected molecular weight (MW) of HoxF ~ 57.8 kDa, HoxU ~ 26.2 kDa, HoxE ~ 18.8 kDa. Ladder, Biorad Precision Plus Protein Dual Color Standards (Catalogue no. 1610374). Gels were stained with Coomassie stain.


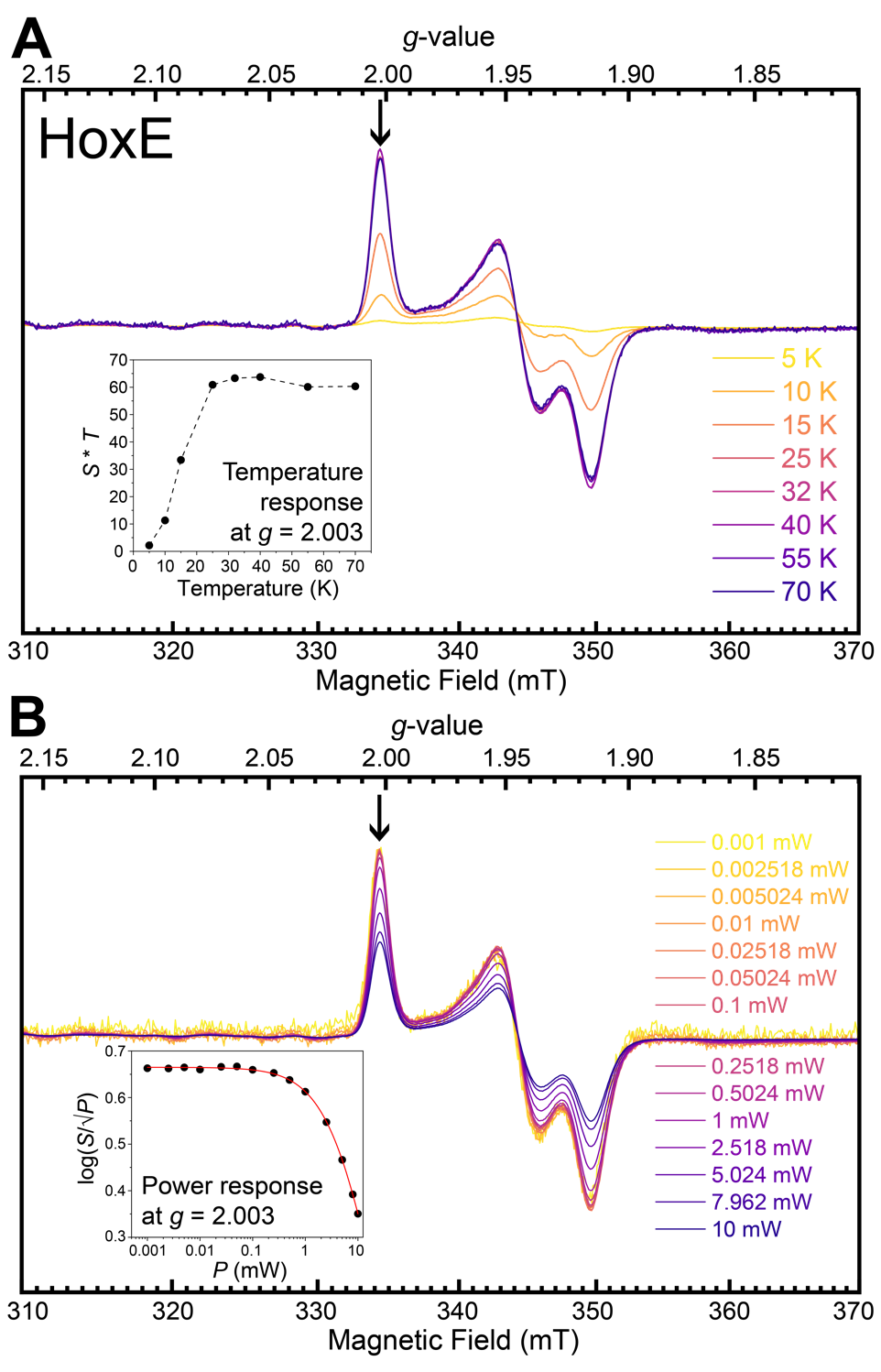


**Figure S2**. The effect of power (*P*) and temperature (*T*) on the EPR signal intensity (*S*) of 50 μM HoxE reduced with 5 mM sodium dithionite. (**A**) Variable temperature data collected using *P* = 0.1 mW and shown scaled to correct for a Curie-like temperature dependence on signal (*S* x *T*). The inset shows a temperature saturation plot monitoring the corrected intensity at *g*_1_ = 2.003 (arrow). Temperature saturation of the signal is readily observed below 25 K; above 25 K the signal displays Curie-like behavior and remains relatively free from temperature broadening effects, with a maximal scaled intensity, or optimal temperature (T_opt_), at 40 K. (**B**) Variable power data collected at *T* = 40 K and shown scaled to correct for signal dependence on power (*S* µ √P). The inset shows a power saturation plot obtained by monitoring the corrected intensity at *g*_1_ = 2.003 (arrow). The power at half-saturation of the signal (*P*_½_) was determined by fitting (red trace) the data (black dots) to **Eqn. 2** as described in the **Methods**, yielding a *P*_1/2_ = 5.0 ± 0.4 and *b* = 1.32 ± 0.07 Small features in the baseline are due to minor manganese contamination.


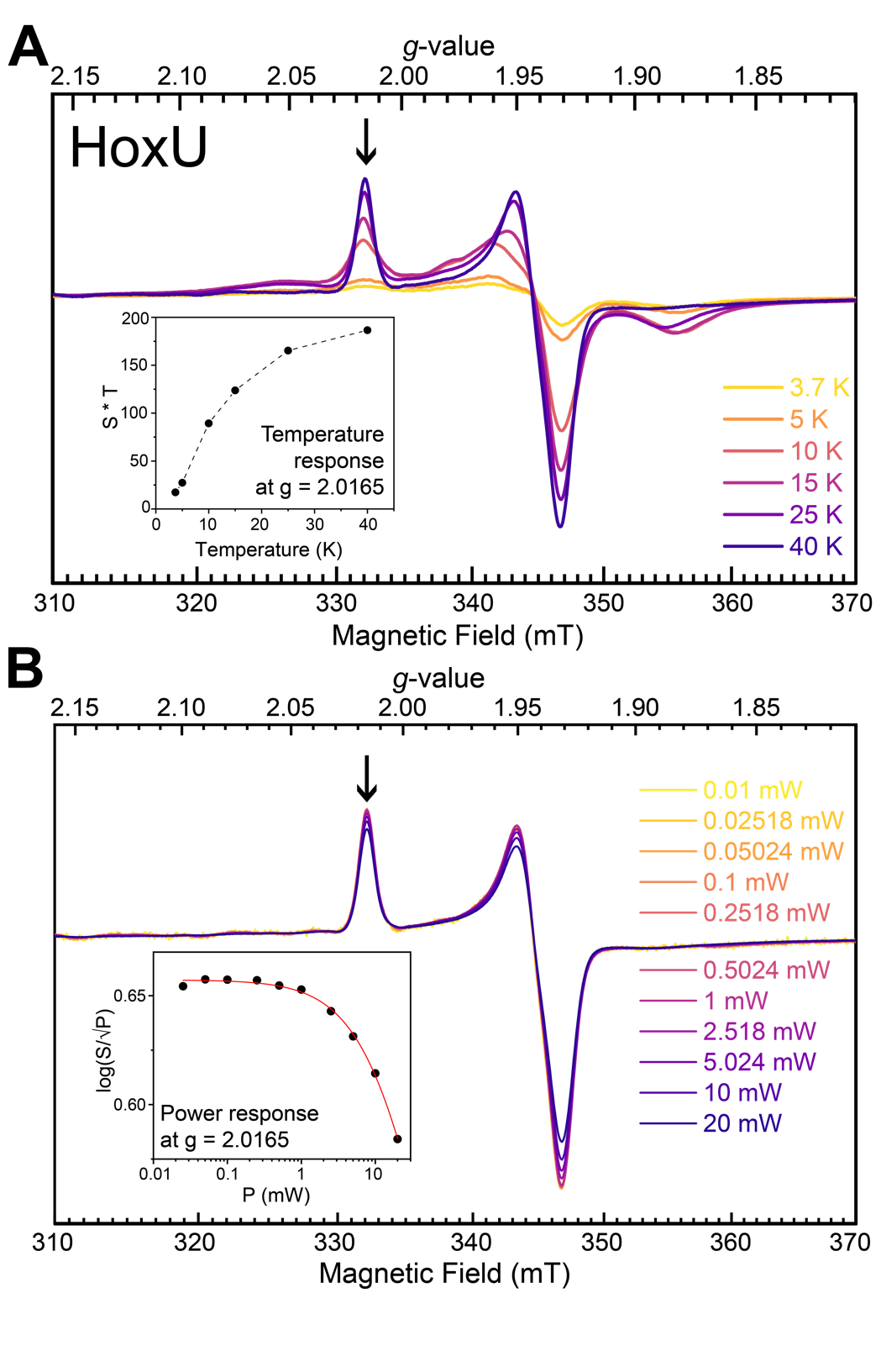


**Figure S3.** The effect of power (*P*) and temperature (*T*) on the EPR signal intensity (*S*) of 50 μM HoxU reduced with 5 mM sodium dithionite. (**A**) Variable temperature data collected using *P* = 1 mW and shown scaled to correct for a Curie-like temperature dependence on signal (*S* x *T*). The inset shows a temperature saturation plot monitoring the corrected intensity at *g*_1_ = 2.0165 (arrow). Temperature saturation of the signal is observed below 40 K with a maximal scaled intensity, or optimal temperature (T_opt_), at 40 K. (**B**) Variable power data collected at *T* = 40 K and shown scaled to correct for signal dependence on power (*S* µ √P). The inset shows a power saturation plot obtained by monitoring the corrected intensity at *g*_1_ = 2.0165 (arrow). The power at half-saturation of the signal (P_½_) was determined by fitting (red trace) the data (black dots) to **Eqn. 2** as described in the **Methods**, yielding a *P*_1/2_ = 14.6 ± 3.7 and *b* = 0.39 ± 0.07. Small features in the baseline are due to minor manganese contamination.


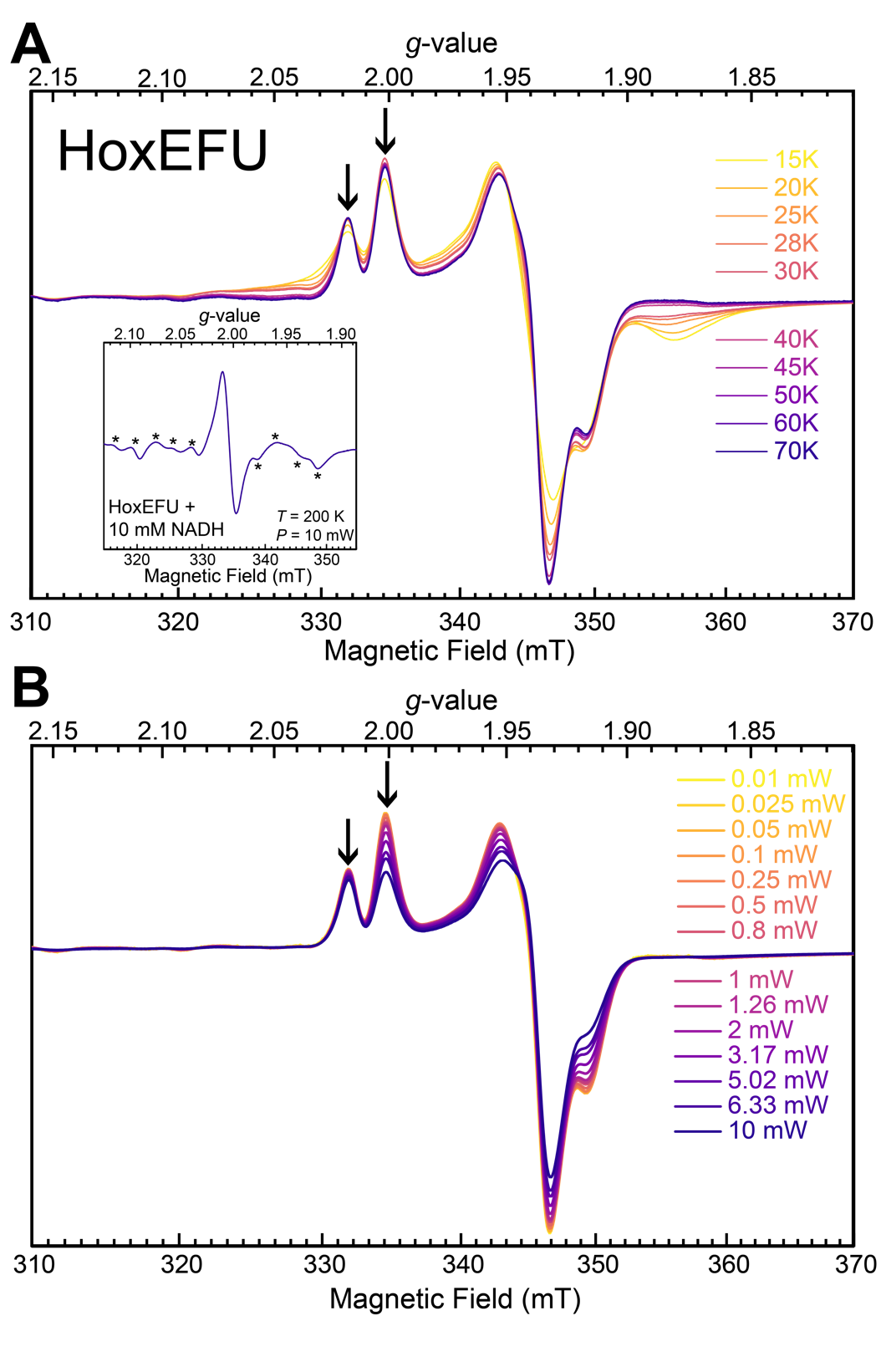


**Figure S4**. The effect of power (*P*) and temperature (*T*) on the EPR signal intensity (*S*) of 50 μM HoxEFU reduced with 5 mM sodium dithionite. (**A**) Variable temperature data collected using *P* = 0.1 mW and shown scaled to correct for a Curie-like temperature dependence on signal (*S* x T). Inset of (**A**) shows data from a sample of HoxEFU reduced with 10 mM NADH (*T* = 200 K; *P* = 10 mW) displaying signal from a flavin radical centered at *g* ~ 2. Features from manganese contamination are indicated with asterisks. (**B**) Variable power data collected at *T* = 40 K and shown scaled to correct for signal dependence on power (*S* µ √P). Arrows indicate spectral features monitored for the HoxU (*g* = 2.0175) and HoxE/F (*g* = 2.002) saturation plots shown in **Figure S5**. Small features in the baseline are due to minor manganese contamination.


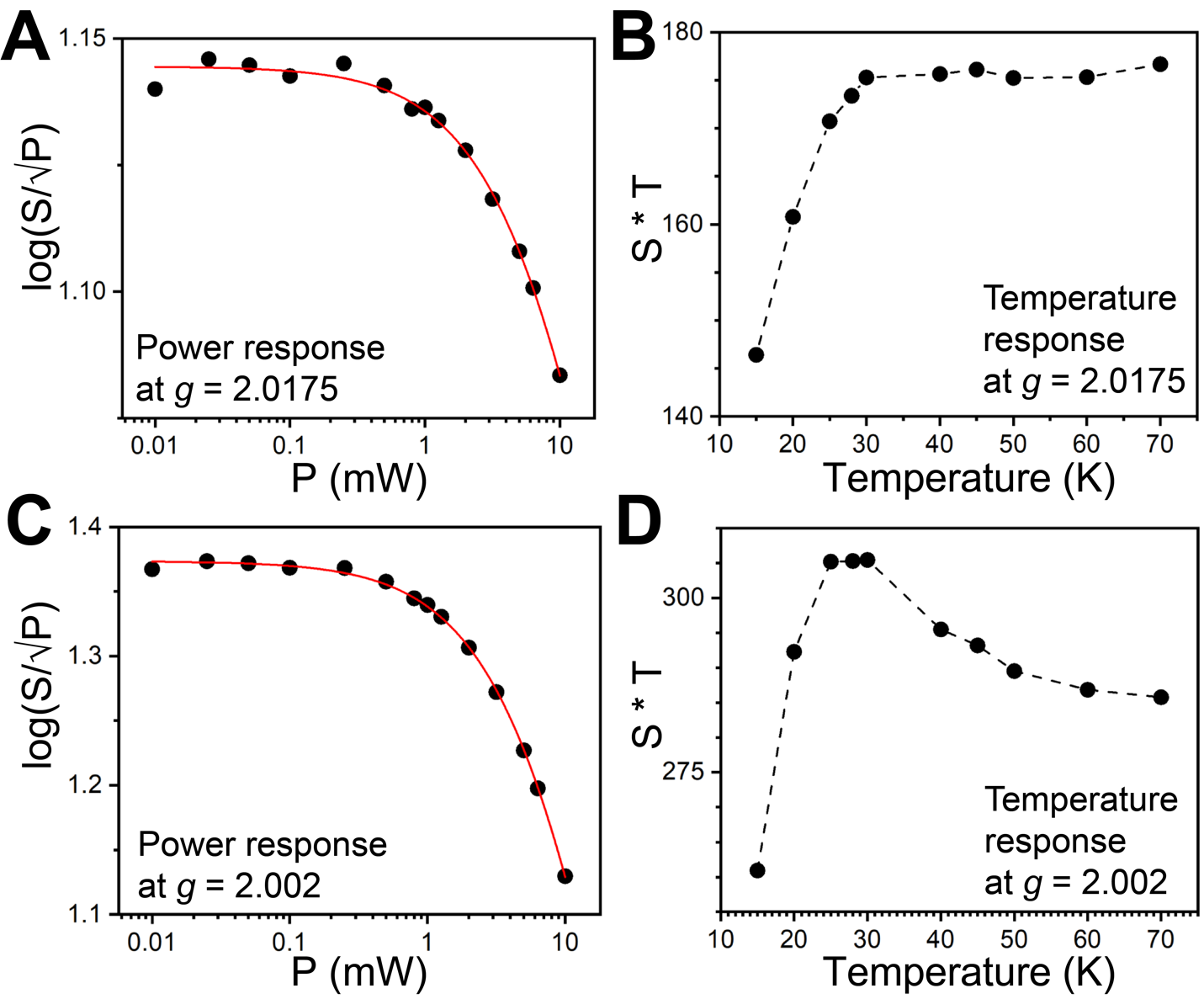


**Figure S5.** Power (left) and temperature (right) saturation curves tracking [2Fe-2S] cluster signals from the HoxEFU complex. Data obtained from spectra shown in **Figure S4** (*T* = 40 K; *P* = 0.1 mW). (**A-B**) The HoxU [2Fe-2S] signal of the complex was monitored at *g* = 2.0175 where there is no overlap with other [2Fe-2S] signals. The power at half-saturation of the signal (P_½_) was determined by fitting (red trace) the data (black dots) to **Eqn. 2** as described in the **Methods**, yielding a *P*_1/2_ = 8.1 ± 2.8 and *b* = 0.35 ± 0.09. Temperature saturation was observed below 30 K with minimal temperature broadening effects observed at higher temperatures. Note this data was obtained from spectra collected with *P* = 0.1 mW rather than the *P* = 1 mW used for variable temperature data of isolated HoxU. (**C-D**) Due to the spectral overlap of the HoxE and HoxF [2Fe-2S] cluster signals they could not be assessed individually and the relaxation behavior of both these clusters is represented in the data collected at *g* ~ 2.002 (i.e., HoxE/F). Monitoring this feature allowed the signal response to be assessed free from contribution of the HoxU [2Fe-2S] cluster. For aid in comparison to isolated HoxE (*P*_1/2_ = 5.0 ± 0.4; *b* = 1.32 ± 0.07), a fit was performed to the power saturation data of HoxE/F yielding a *P*_1/2_ = 8.3 ± 1.0 and *b* = 1.4 ± 0.1. The temperature response was similar to isolated HoxE with temperature saturation below 25 K. Some signal loss is observed above 30 K but this does not appear due to temperature broadening effects as the line-shape of the feature remains the same.


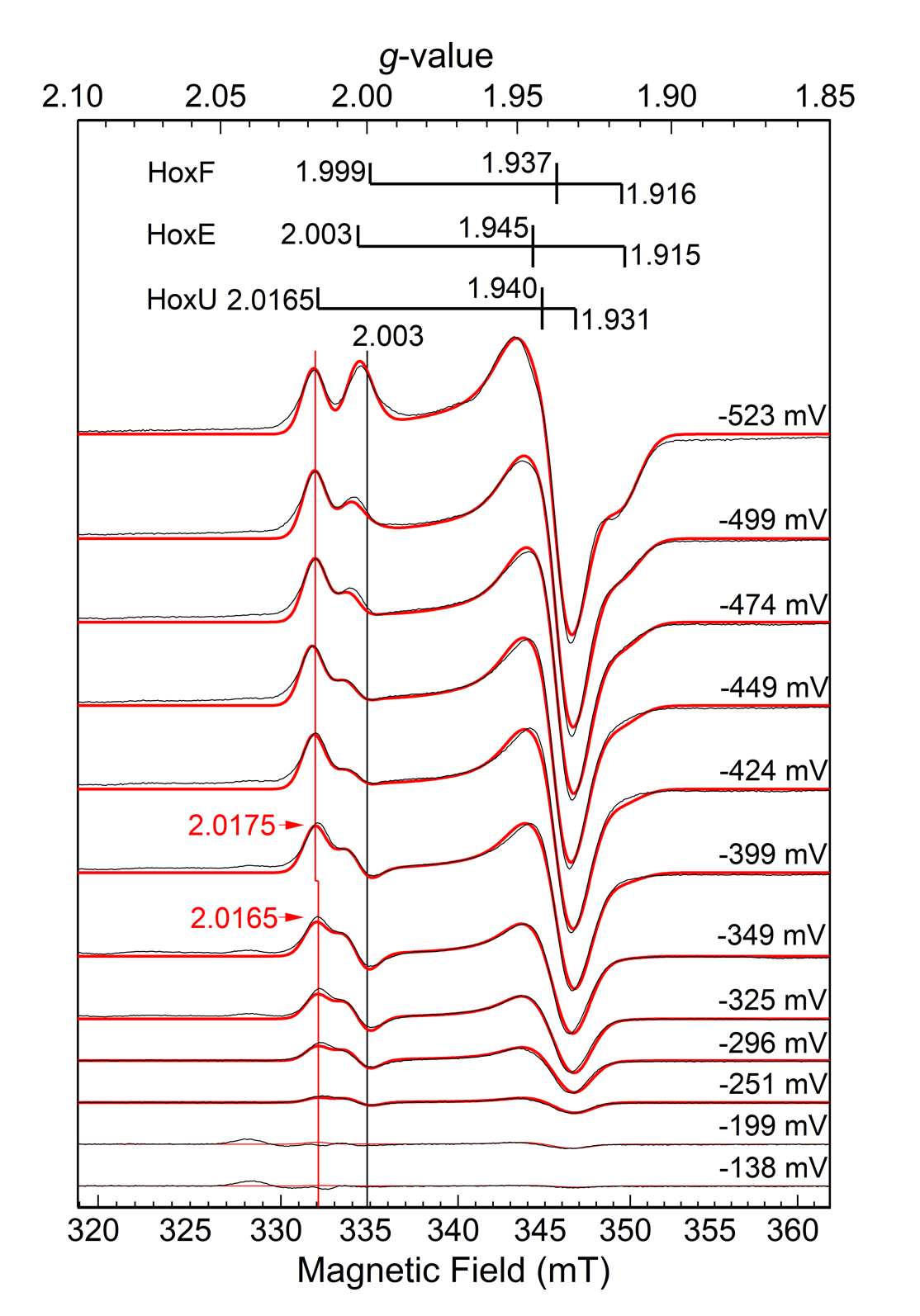


**Figure S6**. Potentiometric EPR data of 50 μM HoxEFU. Raw EPR spectra (black traces) of redox poised samples shown overlaid with simulations (red traces) constructed using parameters given in **Table S1**. Simulations were scaled to the raw spectral intensities and provided a breakdown of the simulated total spectral intensity of the HoxE, HoxF, and HoxU [2Fe-2S] cluster signals throughout the titration. This data was used for construction of the Nernst curves depicted in **Figure 3** and **Figure S7.** All spectra were measured at 40 K and 0.1 mW. The shift of the HoxU [2Fe-2S] cluster *g*_1_ feature is marked by the red line; samples poised at potentials higher than -375 mV displayed only signal from the HoxU [2Fe2S] with a feature simulated at *g*_1_ = 2.0165, while in samples poised at potentials lower than -375 mV this feature shifted downfield and is simulated using *g*_1_ = 2.0175. The peaks from 327 to 335 mT seen in the –199 mV and –138 mV samples are characteristic of the oxidized protein. The vertical black line marks the radical signal at *g* = 2.003 from redox dye mediators used in the titration.


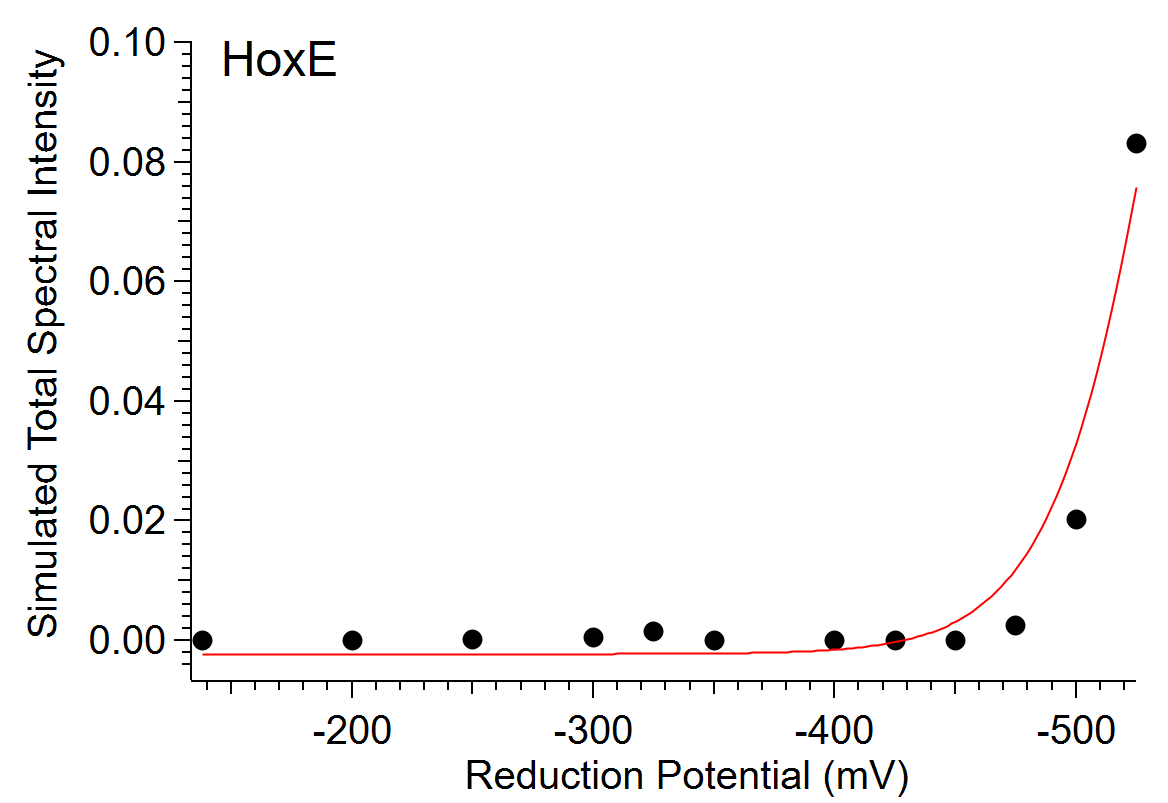

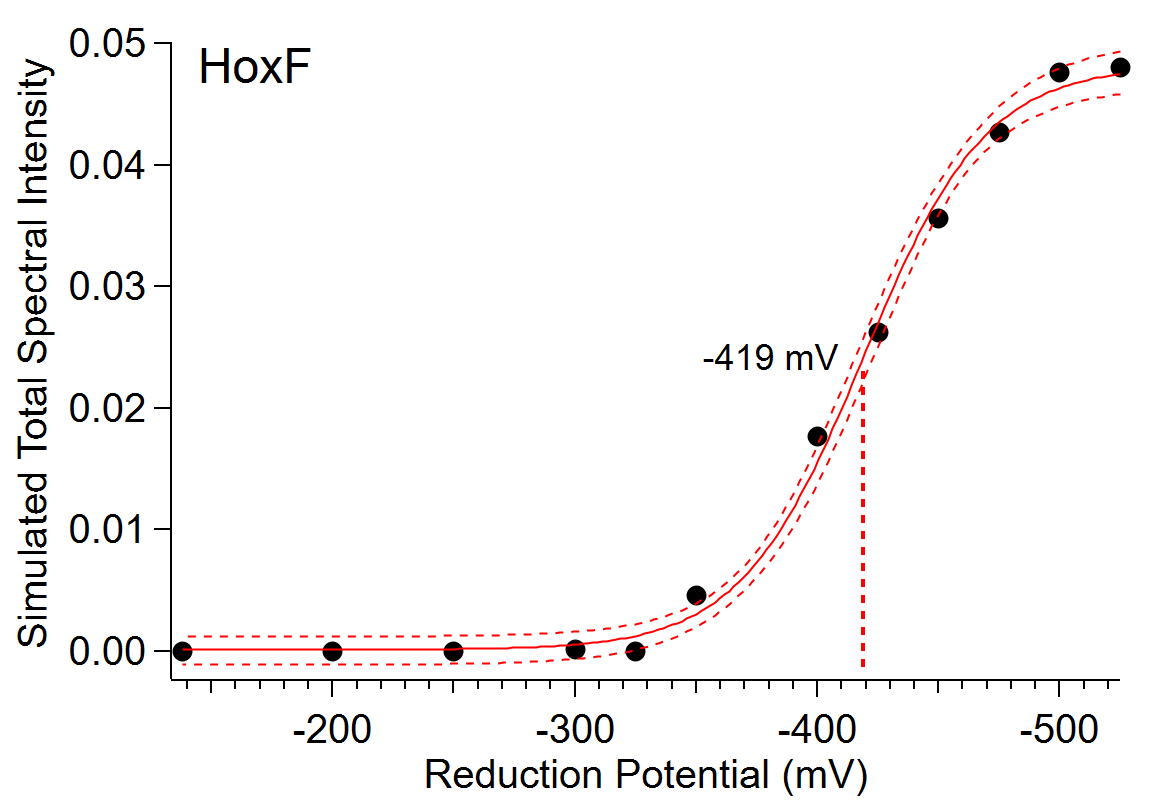

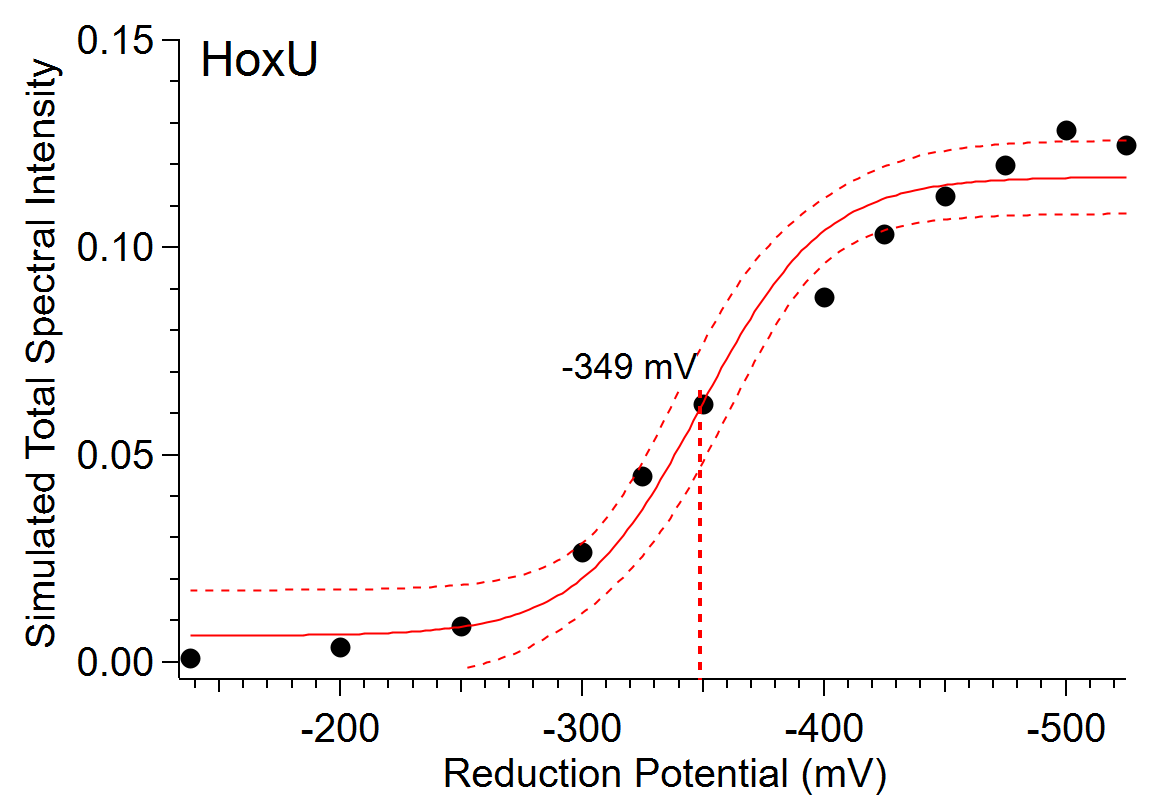


**Figure S7**. Nernst fits (solid red trace) for the [2Fe-2S] clusters of HoxE (*Top*), HoxF (*Middle*), and HoxU (*Bottom*) obtained from simulations of the HoxEFU potentiometric titration spectra seen in **Figure S6**. Black dots represent the simulated total intensity of each [2Fe-2S] cluster spin system at each potential and dashed red lines represent the upper and lower 95% confidence intervals of the fit. The simulated intensity plotted for each potential was fit to the 1-electron Nernst equation (**Eqn. 3**) using IgorPro. The y-axis units are arbitrary units consistent between spin systems and accurately represent the relative contribution of each system at a given reduction potential. Nernstian fit of HoxE data shown for an *E_m_* = - 550 mV.


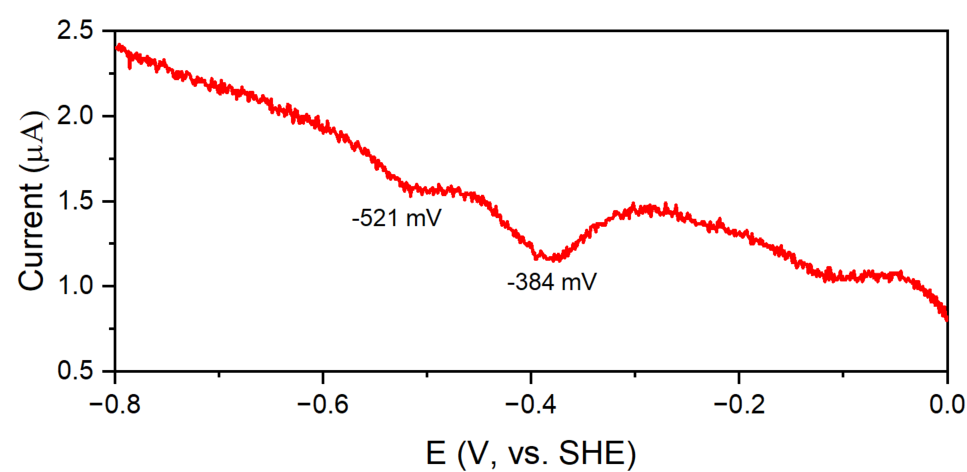


**Figure S8**. Square wave voltammetry of 50 μM HoxEFU collected using a PGE working electrode with the potential (E) scanning in the oxidizing direction. In accordance with the EPR potentiometric titrations, the observed signal at -521 mV can be attributed in part to the [2Fe-2S] cluster found in HoxE. Additional signal at -384 mV likely stems from several of the other [4Fe-4S] and [2Fe-2S] clusters contained within the other subunits of the HoxEFU complex. Voltage (V) indicated versus the Standard Hydrogen Electrode (SHE).


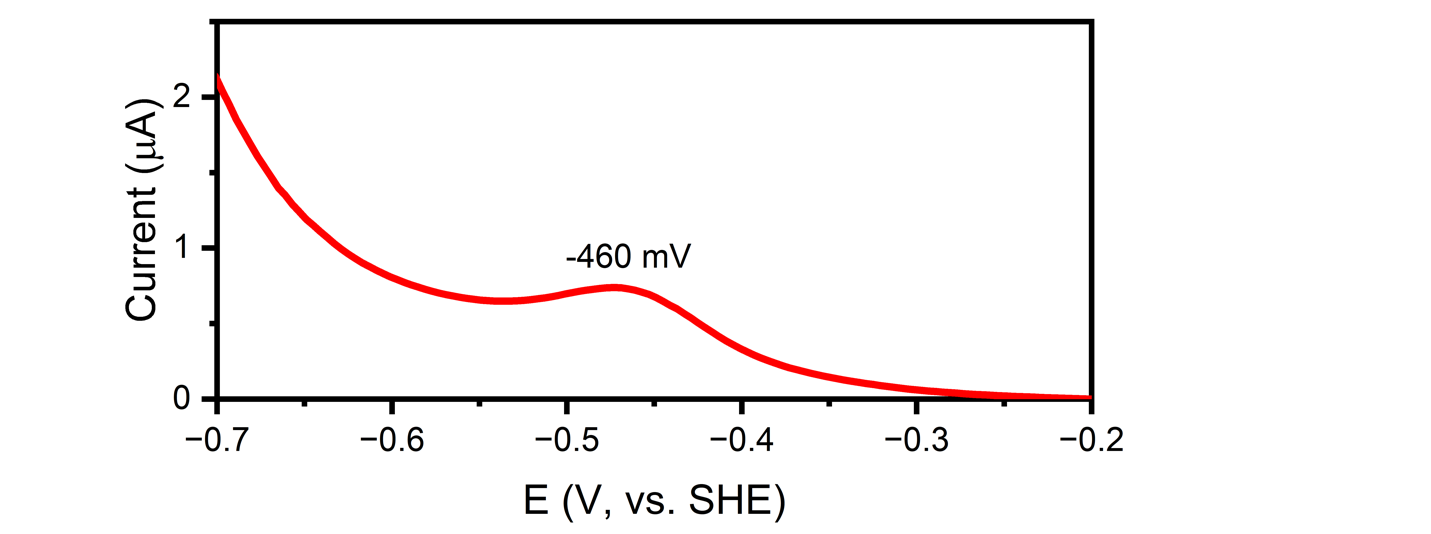


**Figure S9**. Square wave voltammetry of ferredoxin 4 (Fdx4) collected using a gold working electrode, with the potential (E) scanning in the reducing direction. The observed signal at -460 mV is assigned to the *E*_m_ of the [2Fe-2S] cluster of Fdx4. Voltage (V) indicated versus the Standard Hydrogen Electrode (SHE).


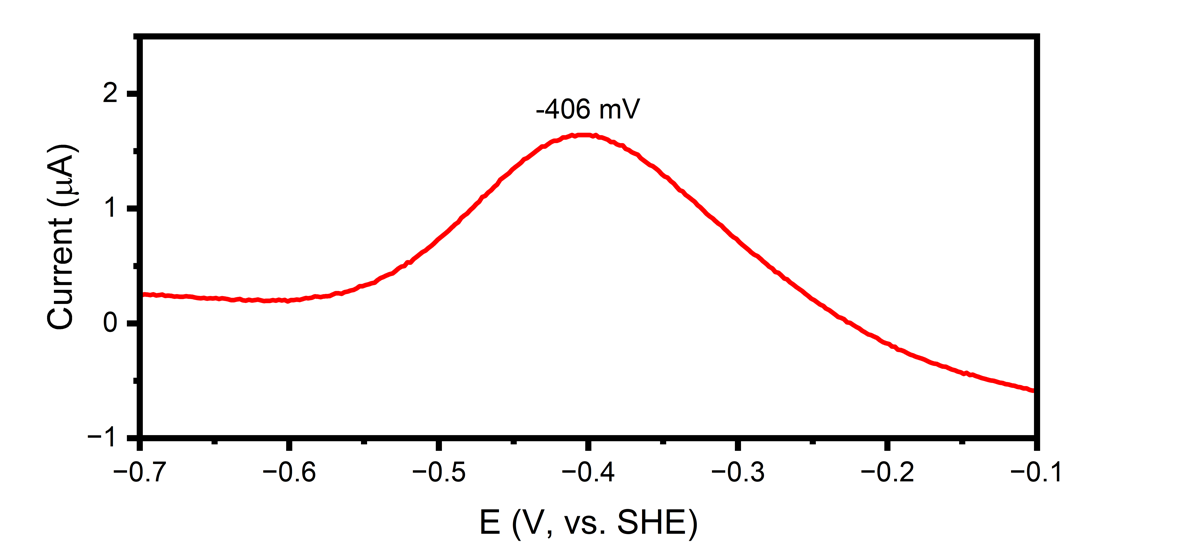


**Figure S10**. Square wave voltammetry of ferredoxin 11 (Fdx11) collected using a PGE working electrode, with the potential (E) scanning in the reducing direction. The observed signal at -406 mV is assigned to the *E*_m_ of the [2Fe-2S] cluster of Fdx11. Voltage (V) indicated versus the Standard Hydrogen Electrode (SHE).

**Table S1**. EPR simulation parameters.

| **Species** | **Sample** | ***g*-values** | ***g-*value strains** | **Figure** |
| --- | --- | --- | --- | --- |
| **HoxE** | HoxEFU titration | 2.003 1.9454 1.9154 | 0.009 0.0144 0.0133 | S6 |
|  | DT reduced  HoxEFU |  | 0.009 0.0156 0.0133 | 2 |
|  | DT reduced  isolated HoxE |  | 0.009 0.016 0.013 | 2 |
| **HoxF** | HoxEFU titration | 1.9993 1.9366 1.9157 | 0.01 0.0079 0.015 | S6 |
|  | DT reduced  HoxEFU |  | 0.01 0.0076 0.015 | 2 |
| **HoxU**^a^ | HoxEFU titration  > -375 mV | 2.0165 1.940 1.931 | 0.009 0.0159 0.013 | S6 |
|  | HoxEFU titration  < -375 mV | 2.0175 1.940 1.931 |  | S6 |
|  | DT reduced  HoxEFU | 2.0175 1.940 1.931 | 0.009 0.01567 0.0117 | 2 |
|  | DT reduced  isolated HoxU | 2.0165 1.944 1.931 | 0.00845 0.01398 0.0117 | 2 |

^a^ Simulations of the HoxEFU data revealed a potential-dependent shifting of the first *g*-value (*g*_1_) for the system assigned to the HoxU [2Fe-2S] cluster, which also correlated with the presence of the HoxE and HoxF [2Fe-2S] clusters. In potentiometric samples poised at potentials positive of -375 mV the HoxU signal alone was present in the spectra, as signified by an isolated feature at *g*_1_ = 2.0165. As potential was lowered below -375 mV this feature shifted downfield (*g*_1_ = 2.0175), concomitant with reduction of the HoxF [2Fe-2S] cluster. No further shifting of this feature was observed with appearance of the HoxE [2Fe-2S] cluster signal at potentials below -475 mV. In the fully reduced HoxEFU data, simulation of the HoxU signal also necessitated the *g*_1_ value of 2.0175 required for the lower potential titration data. This observed “shifting” of the HoxU features may signify an actual change in HoxU *g*-values (e.g., from conformational changes or shifts in cluster microenvironment) and/or may represent modulation of the observed signal due to the presence of spin-spin coupling with a nearby reduced cluster.

**Table S2.** The Fe atom, FMN, and reduced [2Fe-2S] cluster spin content/mol for HoxEFU, HoxU, and HoxE.

| **Protein** | **Expected Fe atoms/mol^a^** | **Measured Fe atoms/mol^b^** | **Expected FMN/mol^c^** | **Measured FMN/mol^d^** | **Expected spins/mol^e^** | **Measured**  **spins/mol^f^** |
| --- | --- | --- | --- | --- | --- | --- |
| HoxEFU | 22 | 20 ± 2 | 1 | 0.98 ± 0.04 | 3 (40 K) | 2.0 (40 K) |
|  |  |  |  |  | 7 (15 K) | 7.3 (15 K) |
| HoxU | 14 | 11 | N/A | N/A | 1 (40 K) | 0.8 (40 K) |
| HoxE | 2 | 1.2 | N/A | N/A | 1 (40 K) | 0.8 (40 K) |

^a^ Expected Fe atoms/mol based on the known cofactors in each subunit or subcomplex. HoxEFU coordinates 4 [4Fe-4S] clusters and 3 [2Fe-2S] clusters for 22 Fe atoms per mol of protein (15 K), HoxU coordinates 3 [4Fe-4S] and 1 [2Fe-2S] clusters for 14 Fe atoms, and HoxE coordinates a single [2Fe-2S] cluster for 2 Fe atoms per mol protein (40 K).

^b^ Fe atoms/mol measured as described previously (1).

^c^ Expected FMN/mol protein based on the known cofactors in each subunit or subcomplex. HoxEFU coordinates a single FMN in the HoxF subunit.

^d^ FMN/mol measured as described previously (2).

^e^Expected spin quantifications. HoxEFU at 15 K, 3x[2Fe-2S] and 4x[4Fe-4S] clusters, 7 spins/mol; HoxEFU at 40 K, 3x[2Fe-2S] clusters, 3 spins/mol; HoxE at 40 K, 1x[2Fe-2S] cluster, 1 spin/mol; HoxU at 40 K, 1x[2Fe-2S] cluster, 1 spin/mol.

^f^Spin quantifications were performed on samples reduced with 5 mM NaDT, pH 8.3. HoxEFU at 15 K and 0.1 mW, HoxEFU and HoxE performed at 40 K and 0.1 mW, and HoxU at 40 K and 1 mW. We note the quantification of HoxEFU was performed at 0.1 mW to avoid saturation of the HoxE and HoxF clusters; however, this power is below the optimal power of HoxU (see Table 1). The breakdown of the spin/mol of HoxE, HoxF and HoxU [2Fe-2S] clusters in HoxEFU are 0.8, 0.4 and 0.8, respectively. See methods for experimental details.

**References**

1. Fish, W. W. (1988) Rapid colorimetric micromethod for the quantitation of complexed iron in biological samples. *Methods Enzymol* **158**, 357-364

2. Wise, C. E., Ledinina, A. E., Mulder, D. W., Chou, K. J., Peters, J. W., King, P. W., and Lubner, C. E. (2022) An uncharacteristically low-potential flavin governs the energy landscape of electron bifurcation. *Proc Natl Acad Sci U S A* **119**, e2117882119
